# How can we better capture food away from Home? Lessons from India’s linking person-level meal and household-level food data

**DOI:** 10.1016/j.foodpol.2017.08.015

**Published:** 2017-10

**Authors:** John L. Fiedler, Suryakant Yadav

**Affiliations:** a The International Dietary Data Expansion (INDDEX) Project, Poverty, Health and Nutrition Division, International Food Policy Research Institute, 2006 K Street NW, Washington, DC 20006, United States; b Department of Development Studies, International Institute of Population Sciences (IIPS), Mumbai 88, India

**Keywords:** Household surveys, Measurement error, Questionnaire design, Food away from home, Dietary assessment, Food security assessment

## Abstract

Despite acknowledged shortcomings, household consumption and expenditure surveys (HCES) are increasingly being used to proxy food consumption because they are relatively more available and affordable than surveys using more precise dietary assessment methods. One of the most common, significant sources of HCES measurement error is their under-estimation of food away from home (FAFH). In 2011, India’s National Survey Sample Organization introduced revisions in its HCES questionnaire that included replacing “cooked meals”—the single item in the food consumption module designed to capture FAFH at the household level—with five more detailed and explicitly FAFH sub-categories. The survey also contained a section with seven, household member-specific questions about meal patterns during the reference period and included three sources of meals away from home (MAFH) that overlapped three of the new FAFH categories.

By providing a conceptual framework with which to organize and consider each household member’s meal pattern throughout the reference period, and breaking down the recalling (or estimating) process into household member-specific responses, we assume the MAFH approach makes the key respondent’s task less memory- and arithmetically-demanding, and thus more accurate than the FAFH household level approach. We use the MAFH estimates as a reference point, and approximate one portion of FAFH measurement error as the differences in MAFH and FAFH estimates. The MAFH estimates reveal marked heterogeneity in intra-household meal patterns, reflecting the complexity of the HCES’s key informant task of reporting household level data, and underscoring its importance as a source of measurement error.

We find the household level-based estimates of FAFH increase from just 60.4% of the individual-based estimates in the round prior to the questionnaire modifications to 96.7% after the changes. We conclude that the MFAH-FAFH linked approach substantially reduced FAFH measurement error in India. The approach has wider applicability in global efforts to improve HCES.

## Highlights

• For decades, India’s HCES has collected individual household member data on meals.
• Underreported food away from home had been a growing source of measurement error.
• Household level food questions linked to members’ meals questions were introduced.
• The changes markedly reduced discrepancies between FAFH and meals away from home.
• More countries should introduce similar changes to improve food security measures.

## 1 Introduction

Understanding diets and designing and monitoring effective food and nutrition programs and policies requires food consumption data. While 24 h recall and observed-weighed food records are widely regarded as the most precise dietary assessment methods, their technical and resource requirements put these methods out of the reach of most countries. Relatively few surveys employ these methods, and those that have are generally small and not nationally representative. The resulting food consumption information gap has given rise to the growing use of Household Consumption and Expenditure Surveys (HCES).

The use of HCES data to proxy food consumption has grown steadily over the past 25 years despite the fact that they have acknowledged shortcomings (Fiedler, 2013). Their use has grown because they are relevant, available, accessible and affordable: they contain a great deal of information about food acquisition and consumption; are conducted routinely in most countries; are statistically representative at national and sub-national levels; and are already being paid for by government. Although HCES vary substantially by country, most share some common shortcomings stemming from the design of their questionnaires (Fiedler et al., 2012).^3^ For this analysis of specifically the food and nutrition data collected by HCES, the most common question-related shortcomings are that they:

• consist of household-level—not individual-level—data,
• contain a mixture of consumption and purchases,
• include unstandardized units for reporting food volume or weight,
• do not adjust for food that is wasted, given to animals or given to persons who are not household members and
• they inadequately capture food that is prepared and consumed outside of the home (Smith et al., 2014).

This paper analyzes measurement error due to questionnaire design, focusing on just the food consumption and expenditures data of HCES and in particular on food away from home. HCES have been conducted in low- and middle-income countries for more than half a century. HCES collect data using either diaries or household interviews, with interviews being the most common approach, roughly 70%. The typical interview form collects food expenditure data using a close-ended list of roughly 100 common food items. The early HCES questionnaires included relatively short lists of commodity-like foods, much like the FAO’s Food Balance Sheets. Over time, countries have added increasing numbers of processed foods, but it is only in the last decade or two that most have even begun to ask about FAFH. As eating away from home has become increasingly common worldwide, countries have come to recognize that adequately capturing food expenditures requires collecting accurate data on FAFH. A recent review of the HCES of 100 countries, found that 90% of countries now collect some information about FAFH. The ways in which they do so, however, vary substantially, reflecting the fact that most of the approaches are “*ad hoc* and unsatisfactory” (Smith et al., 2014). Few have been carefully empirically assessed and to our knowledge there has never been an experiment or a comparative analysis of the strengths and weaknesses of alternative approaches.

While it is widely recognized that FAFH is subject to considerable measurement error, just how much it contributes to under-estimating consumption is unknown. However, in light of the fact that FAFH is expected to continue to grow as a proportion of both total food consumed and total food expenditures, absent change in how information is collected, the magnitude of that under-estimation can be expected to increase. As it does, it will exacerbate the instability of HCES-based estimates of food insecurity and under-nutrition as currently measured, (Tandon and Landes, 2011, D’Souza and Tandon, 2014, Smith, 2015), obfuscate trends and prompt more to question whether even the general order of magnitude of our estimates of global under-nutrition should be accepted (Banerjee and Duflo, 2011). The inadequate collection of FAFH data urgently needs to be better understood and systematically improved.

While there is little argument about the importance of FAFH, or that its prevalence and significance are increasing, there remains a paucity of knowledge about even its major characteristics. More fundamentally, there is little discussion about how it should be defined. As its title suggests, FAFH is commonly defined by where food is consumed, regardless of where it is prepared. In other instances, it is defined by where food is prepared, regardless of where it is consumed; in which case, food that is prepared at home and taken outside of the home to be eaten—at work or at school, for instance—is considered FAFH. In this paper, we define FAFH to include only one of the four possible combinations of where food is prepared and consumed; i.e., it includes only food that is prepared and consumed away from home. Food prepared at home and consumed at home, food prepared at home and consumed away and food prepared away and consumed at home, are all captured in home consumption and expenditures data, and as such are not FAFH.

There are other sources of HCES measurement error in FAFH as well, beyond the ambiguities of definition and the limited number and diverse nature of questions asked about it. HCES commonly rely on a single, key respondent to report all household members’ food consumption and expenditures. Food away from home becoming more common has complicated the key respondent’s task of accurately reporting household consumption: the key respondent is increasingly unlikely to be aware of events that occur outside of his/her purview. Larger households, and especially those with larger numbers of adults, persons living in urban areas, those with more complex lifestyles and greater physical mobility, and persons who commonly spend more than 24 h away from home, are particularly likely to have their consumption under-reported. Additional sources of measurement problems associated with FAFH include the need to capture multiple foods from multiple places with different menus and prices, the challenge of estimating the quantity of the different types of foods contained in processed foods, and how much of it was consumed. No doubt, this is challenging work and measurement error will never be eliminated. The relevant question is, how can it be reduced: how can we improve the measurement of FAFH in HCES?

## 2 Revisiting the great Indian calorie debate

Over the past decade, a lively debate has waged over the seemingly paradoxical National Survey Sample Organization- (NSSO-) based finding that India’s income and its middle class have grown dramatically since the late 1980 s, while its per capita caloric consumption has fallen. Explanations have been wide-ranging:

• Indians have less need for food: the average calorie requirements of Indians have fallen due to increased mechanization and relatively greater growth in less strenuous physical work (Rao, 2000, Deaton and Dreze, 2009, Eli and Li, 2012).
• Poverty has been inaccurately measured and its prevalence has actually increased (Patnaik, 2010).
• Indians are opting to spend relatively more of their income on non-food items (Banerjee and Duflo, 2011).
• There has been a food budget squeeze: general food demand has fallen due to increasing food prices (Patnaik, 2010).
• The relative cost of fuel in rural India has increased and crowded-out food (Basole and Basu, 2014).
• While it should be recognized that the NSS was not originally designed to be used for comprehensive food security analysis, it should be used to do so, and would do a better job if “some minor amendments in food data collection, particularly in respect of the part of food consumed outside home (and incorporating the) refuse factor of food items” (Chattapadhyay et al., 2010).
• Calorie consumption is inadequately measured by the NSS primarily due to the inadequate capturing of FAFH (Smith, 2015).

FAFH behaviors are highly variable: they vary by household composition and income, and the determinants of occasional eating out have been found to be distinct from those of persons who eat out more regularly (Naska et al., 2015, Liu et al., 2015, Orfanos et al., 2013). Among people for whom FAFH is an occasional behavior, it is not easy to determine whether the days that individuals or households do not report eating out reflect their usual habits or not. Depending upon the frequency of “usual” eating out patterns, the length of the recall period may exert undue influence on the estimates, and if too short will result in unstable estimates. This suggests that to be better able to design a survey to capture FAFH there is a need to better understand the frequency and general nature of patterns of FAFH. One common approach in attempting to develop a better understanding of eating away from home is to ask about the place of consumption. A number of studies have found the location of out of home consumption to be systematically related to the probability of consuming food away from home, the frequency of out-of-home consumption, in the composition of foods consumed and nutrient intakes (Naska et al., 2015, Liu et al., 2015, Orfanos et al., 2007, Orfanos et al., 2009, Vandevijvere et al., 2009).

**Fig. 1:**
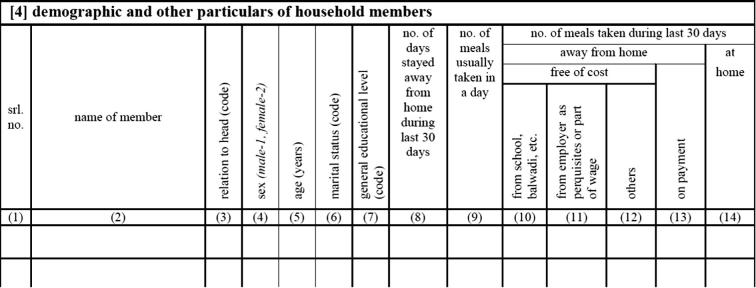
The Indian HCES household roster Block 4 meal questions.

For more than three decades, the Indian HCES questionnaire has included a seven-question section that asks about the number and type of meals consumed by each individual household member (Fig. 1). The responses to these questions provide the wherewithal for developing a detailed understanding of the composition of meals away from home (MAFH) and meal consumption patterns. Although the place of FAFH is not identified, distinct types of meals are, and may provide similar insights into systematic differences in individuals’ and households’ FAFH, which in turn can be helpful in devising ways to better capture FAFH.^4^

This study analyzes the most recent quinquennial survey, the 68th round, 2011–2012, and combines the analysis of MAFH with the introduction of modifications in the food list designed to be better able to understand and measure FAFH and to more definitively unravel the apparent paradox of falling average caloric intakes and rising average income. The objectives of the paper are threefold: (1) investigate the potential usefulness of collecting individual household member meal data to better understand FAFH, (2) juxtapose MAFH and FAFH measures to gauge one component of Round 68 FAFH measurement error, and (3) assess the impact of the questionnaire changes on FAFH measurement error.

## 3 Data and methods

### 3.1 The database: 2011–2012 national survey sample Round 68 (NSS-R68)

Since 1950, the Indian National Sample Survey Office (NSSO) has conducted national household surveys. It conducts a continuous socio-economic survey using what is referred to as a “thin” sample, and approximately once every 5 years it conducts a national household consumer expenditure and employment survey based on a “thick” sample. The quinquennial surveys are designed to collect detailed information on the quantity and value of household consumption with the aim of developing estimates of average household monthly per capita consumer expenditures and the distribution of those expenditures by rural–urban sector, socio-economic groups and by state and union territory (SUT).

The sample of the 68th round is a stratified multi-stage design (NSSO, 2014). The first stage units are the 2001 census villages in the rural sector and the urban frame survey blocks in the urban sector. Within each district of a state or union territory (SUT), rural and urban strata were formed. The sample was allocated to the SUTs in proportion to population, subject to a minimum sample allocation for each SUT. The survey was conducted over a period of one year, from July 1, 2011 to June 30, 2012. Four sub-rounds of three months’ duration each were undertaken. First Stage Units (FSUs) were visited in each sub-round to interview roughly one quarter of each FSUs’ sample so as to ensure spreading the sample FSUs over the entire period and enabling the capturing of seasonal variations. The sample provides estimates that are representative at the national level and for each of India’s 35 states and union territories. Sample weights, adjusted for non-response, were included in the database and were used to determine total population estimates of households and persons reported here.

### 3.2 An overview of the study design

In 2011, the NSSO introduced a number of revisions in its HCES questionnaire. It modified the food list by unpacking a single, general household level category of “cooked meals” into five more specific sub-categories. Three of those new household level food categories were identical to three individual household-member-level categories of meals consumed away from home that were asked about in the household roster section of the questionnaire. It was assumed that to the extent that there would be discrepancies in the two sets of numbers, that the individual, household-member-specific MAFA data would be more accurate than the household level FAFH, owing to the considerably more demanding cognitive process involved in the respondent having to calculate and then sum the number of meals for each individual household member; a task that we assumed would be relatively more demanding and to vary by various household characteristics (as discussed earlier). We therefore assumed that the individual household member-specific measures of MAFH would be closer to the “truth” than the household level FAFH questions (i.e., that the individual meals data would more accurately capture the behavior of household members); and we further assumed that the difference in the two measures would provide a partial indication of the (remaining) measurement error in the household level-reported FAFH. To assess the impact of the changes in the questionnaire, we analyzed the data from the previous HCES (Round 64 from 2007/08) and compared the variation in its FAFH and MAFH estimates to those in 2011/12. We hypothesized that the differences in the FAFH-MAFH estimates of the more recent survey would be less because we assumed (again using the MAFH as a gold standard) that the changes introduced would reduce measurement error in FAFH.

### 3.3 The changes in the HCES questionnaire

The 2011/12 Round 68 questionnaire contains three different sets of questions about meals. One of the sets of questions (Fig. 1) is designed to develop an understanding of each household members’ usual daily meal consumption pattern over the last 30 days. It consists of a series of seven questions about the number of meals usually consumed in a day, whether or not each individual household member was away from home for more than 24 h, the number and sources (or types) of meals individual household members ate away from home and the number they ate at home. It asks specifically about four categories of MAFH; meals obtained at school, from employers, from others (other households and government- as well as NGO-related programs) and meals received on payment.^5^

The responses to the seven questions together constitute a unifying conceptual framework that constitutes a memory aid sensitizing both the respondent and the interviewer to each individual household member’s pattern of meal consumption and consumption of FAFH during the reference period, and which provides a tool with which the respondent and the interviewer, alike, are able to perform various consistency checks across the different numbers and types of meals consumed.

**Fig. 2:**
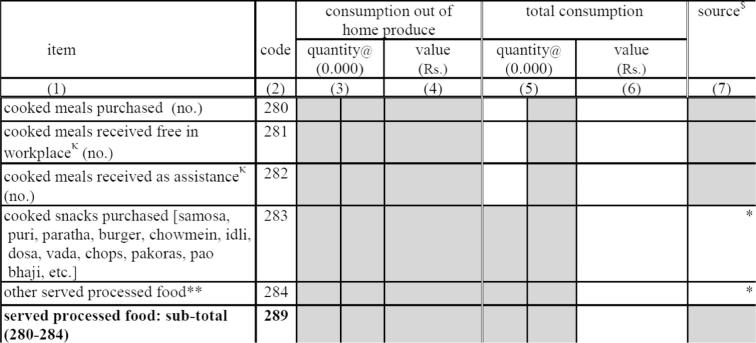
The Indian HCES Block 5 questions introduced in 2011/12, Round 68.

The second section asking about meals is part of the food consumption module’s food list-related questions. The respondent is asked the quantity and the value of the household’s consumption during the past 30 days of each of the items in the questionnaire’s 132 item food list. Prior to 2011/12, there were two items, “cooked meals received from as assistance or payment” and “cooked meals purchased,” on the food list that captured food away from home. In Round 68, these two items were replaced with the five shown in Fig. 2.

**Fig. 3:**
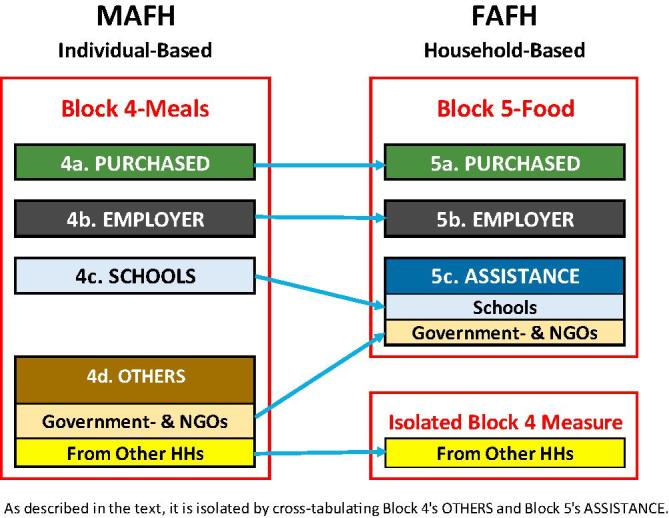
Matching meal away from home and food away from home data in the Indian 2011/12 HCES.

The first three of these new categories of food items are partially redundant with the MAFH data from Block 4: both include Meals Purchased and Meals Received Free-at-Workplace (Free from Employer). They both also include other categories that they define differently but that are partially overlapping (Fig. 3). Block 4 includes two variables, meals received free of cost from SCHOOLS and meals received free of cost from OTHERS, while the Block 5 includes meals received as ASSISTANCE, which also includes schools and other government- and NGO-programs, but (as noted in a footnote in the questionnaire) does not include cooked meals received from other households.^6^ As a result, the difference then between Block 4’s OTHERS plus SCHOOLS and Block 5’s ASSISTANCE is meals received from other households, which is included in the former, but not the latter.

It is possible, however, to develop an estimate of (only) the meals received from other households, so that the MAFH totals can be directly compared to the FAFH totals and we can develop a more comprehensive, less measurement error-plagued, estimate of food away from home. By combining the Block 4 Schools and Others variable and cross-tabulating it with Block 5’s Assistance, we are able to isolate the households that are in the new Block 4 variable, but not in Block 5’s Assistance: these are the meals provided by other households. How well we are able to isolate and identify these households depends on how consistently households reported their MAFH and FAFH. Adding these households and the meals they consumed to the Block 5 provides an estimate of the total number of households that consumed some FAFH in the last 30 days and the total number of meals consumed as FAFH during that period. The estimates of total number of MAFH and FAFH should both be comprehensive and all-inclusive, and they should be equal. We expect that the two approaches will not generate exactly equivalent estimates, and we assume that the household member-specific measures of Block 4 will be more accurate than Block 5’s household level measures. We regard the person-level estimates to be our reference point, a type of “gold standard” for field-based measurement of meals consumed away from home, which constitutes a substantial portion of total FAFH.^7^ We assume that variations in the two estimates are one source of measurement error in FAFH, and we estimate its magnitude.

We also conduct a comparative analysis of the difference in the variations in MAFH and FAFH estimates in Round 64—i.e., a survey found prior to the revision in the questionnaire—with the variations in MAFH and FAFH estimates in Round 68—the first quinquennial survey after the change in the questionnaire—to provide an approximation of the general order of magnitude by which the under-estimation of FAFH has been reduced as a result of the revisions in the questionnaire. Despite our understanding that the changes in the food list items were motivated by recognition of the needs to better capture FAFH and to promote greater consistency in the responses to the Block 4 meals questions and the Block 5 food questions, to our knowledge no such comparative analysis has apparently been done before this one.

## 4 Findings

### 4.1 The numbers and sources of meals eaten in the past 30 days: The block 4 meal questions

**Fig. 4:**
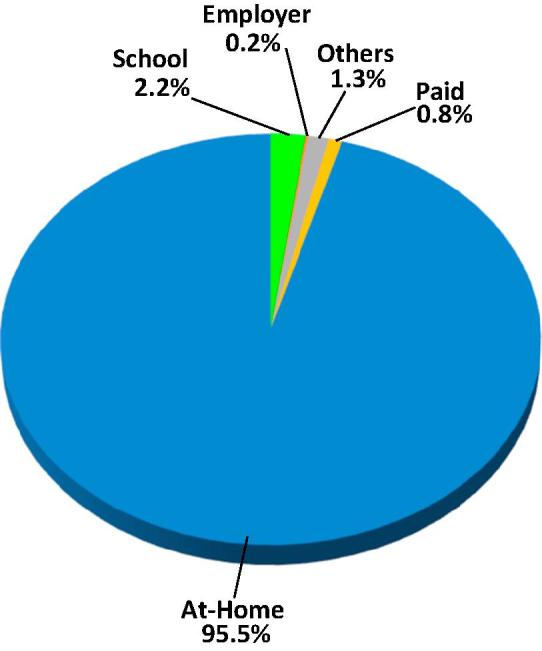
Composition of meals in the last 30 days Block 4, individual household member-specific data.

Over the 30 days prior to being interviewed, Indians on average consumed a mean of 2.5 and a median of 2.0 meals per day. As shown in Fig. 4, the vast majority of meals, 95.5%, were consumed at home. The 4.5% of meals consumed away from home were comprised of meals received from school, employers, others (defined as other households, government-agency- and NGO-sponsored programs) and on payment. While they accounted for less than one in twenty meals, nearly one in every five Indians had at least one such meal.

The conditional average—that is, the average among persons who had at least one meal away from home—was 16.7 (Table 1). Of the four types of meals away from home, school meals were the most common, accounting for nearly half of the total, and 2.2% of all meals. 8.6% of all Indians were reported to have consumed a school meal in the previous 30 days. A slightly larger share, 8.8%, of Indians reported that they had received a meal from “others” in the previous 30 days. The mean number of “other” meals received by those with at least one was 11.0, just 60% of the school meals’ mean of 18.8.

**Table 1:** Individual meal consumption in the last 30 days, India 2011–2012. Unconditional and conditional averages and total numbers by meal type.

Measure	A	B	C	D	E	F	G	H	I
	Number of meals usually taken in a day-last 30 Days	Number of days stayed away from home last 30 days	*Number of meals taken in the last 30* d*ays from:*						
			School	Employer	Others	On Payment	Total Away from Home (= C + D+E + F)	At Home	Total Meals = Away+At Home (= G + H)
*Unconditional*									
No. of Persons Responding	1,10,61,31,724	1,10,61,31,724	1,10,61,31,724	1,10,61,31,724	1,10,61,31,724	1,10,61,31,724	1,10,61,31,724	1,10,61,31,724	1,10,61,31,724
Mean No. of Meals	2.5	0.4	1.6	0.1	1.0	0.6	3.3	70.6	73.9
Median No. of Meals	2.0	0.0	0.0	0.0	0.0	0.0	0.0	60.0	60.0
Total number of Meals	2,73,22,29,898	46,31,82,636	1,79,16,65,714	13,10,06,979	1,06,80,39,644	67,36,59,539	3,66,43,71,875	78,11,69,04,859	81,78,12,76,734
Percent of meals			2.2%	0.2%	1.3%	0.8%	4.5%	95.5%	100.0%

***Percent of persons with a non-zero response***		**7.8%**	**8.6%**	**0.5%**	**8.8%**	**3.6%**	**19.9%**	**99.1%**	**100.0%**

*Conditional*									
No. of Persons with > 0		8,68,14,614	9,53,59,945	53,95,769	9,70,86,831	3,94,10,540	21,97,57,465	1,08,23,21,098	1,10,61,31,724
Mean No. of Meals		5.3	18.8	24.3	11.0	17.1	16.7	72.2	73.9
Median No. of Meals		3.0	20.0	20.0	6.0	6.0	15.0	60.0	60.0
Total number		46,31,82,636	1,79,16,65,714	13,10,06,979	1,06,80,39,644	67,36,59,539	3,66,43,71,875	78,11,69,04,859	81,78,12,76,734
Percent of MAFH			48.9%	3.6%	29.1%	18.4%	100.0%		

Unconditional: Includes all persons, those reporting not consuming any of the meal type indicated and those consuming one or more. Conditional: Includes only persons with one or more meals of the type indicated.

Only 3.6% of Indians reportedly consumed a meal for which they paid (wholly or in part). Purchased meals accounted for only 0.8% of all meals consumed and 18.4% of all meals away from home. Of the four types of meals away from home, those provided by an employer were the least quantitatively significant in terms of the proportion of persons eating one or more, their share of all meals or their share of all meals away from home. However, employer-provided meals had the highest conditional averages of the four types of meals away from home. Its mean was 24.3, about one-third again greater than school meals’ 18.8 or paid meals’ 17.1. For all three of these meal types, persons who received one or more in the past 30 days, received them at a rate of 5–6 days per week, probably a reflection of the common schedule of schools and workplaces. In contrast, persons who had at least one “others” meal generally had roughly one-third fewer such meals, 11. The consumption patterns of the four different MAFHs are highly segmented. Of the Indians who had at least one MAFH, 92% reporting having only one type of MAFH. Among all Indians less than 1.5% reporting having two or more sources of MAFH.

**Fig. 5:**
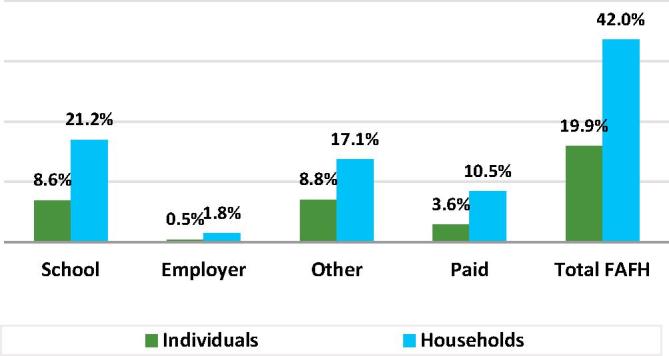
Percent of individuals and households consuming at least one meal away from home, India 2011/12.

As shown in Fig. 5, very different profiles of FAFH in India emerge depending on whether the measures being used to characterize it are household-based or individual household member-based. While 42% of Indian households had at least one meal away from home in the previous 30 days, less than half that number, 19.9%, of all individual Indians had one or more meal away from home. The overall (unconditional) average number of meals consumed outside the home was 14.6 per household and 3.3 per Indian. The conditional means were 34.9 and 16.7, respectively. These differences in the individual versus household-based measures reflect the fact that the distribution of meals away from home varies substantially by individuals within the same household. This intra-household variability is one of the factors accounting for the sensitivity of food security measures to alternative estimation methods that Tandon and others have demonstrated in the case of India, as well as other countries (Tandon and Landes, 2011, D’Souza and Tandon, 2014).

**Fig. 6:**
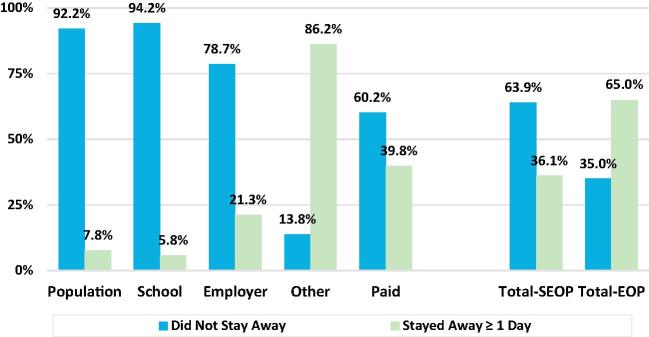
Relative shares of meals away from home by stayed away from home status (last 30 days) individual household member data, India 2011/12.

As shown in Fig. 6, the 7.8% of persons who stayed away from home at least one day in the previous 30 days (defined as a continuous absence from home for 24 h), accounted for a disproportionately large share of three of the four types of MAFH; the exception being school meals. Their share of total MAFH, exclusive of schools (labeled “Total-EOP” in Fig. 6) was roughly twice their share of inclusive of schools (labeled “Total-SEOP”), reflecting the fact that the vast majority of school meals were provided to students living at home.

**Fig. 7:**
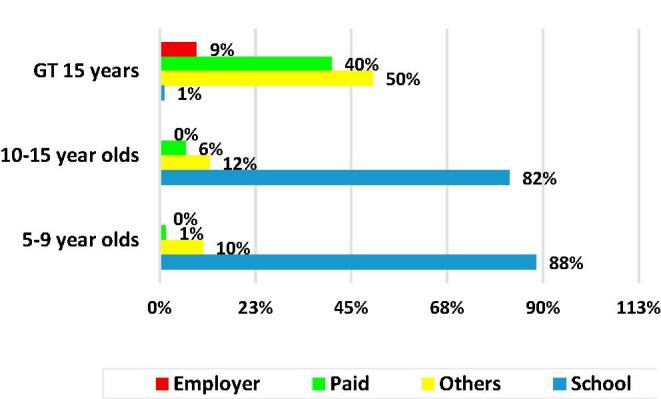
Percent of all meals away from home, by type and age group.

**Fig. 8:**
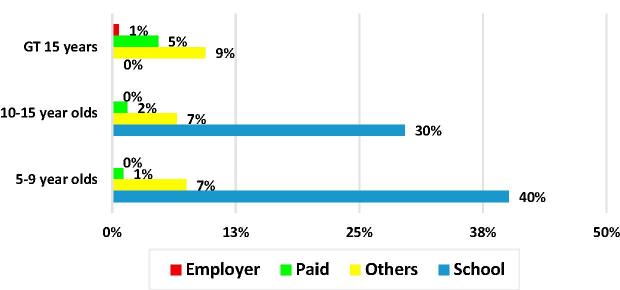
Percent of persons with at least one meal away from home, by type of meal and age group.

Two distinct general typologies of MAFH consumers can be identified. One consists of the primary school children and the second consists of persons 15 years of age and older. The first typology is comprised of primary school children 5–9 years old and upper primary school children 10–15 years old who receive free school meals, whose school meals constitute 88% and 82%, respectively, of all of the MAFH consumed by these age groups (Fig. 7). 88% of the children who were reported to have received one or more school meal, live in rural areas. As shown in Fig. 8, nationwide, the coverage of free school meals was 40% of 5–9 year olds and 30% of 10–15 year olds. In the previous 30 days, children who had at least one school meal, had an average of 19 meals.

**Fig. 9:**
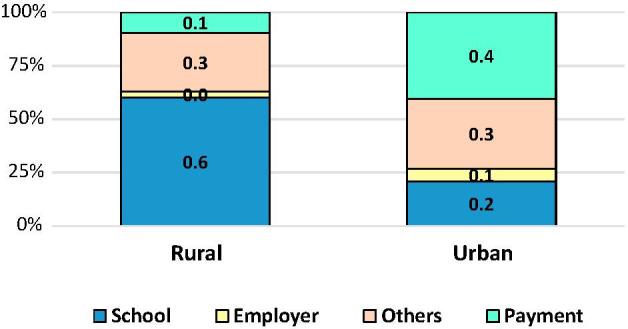
Composition of meals away from home by rural-urban residence, India 2011/12.

The second general typology of FAFH consumers consists of persons report consuming almost only paid and other meals. Persons within this group are nearly twice as likely to have consumed an “other meal” relative to a paid one (Fig. 8) and overall consumed slightly more “other” meals than paid meals (Fig. 7). Males are somewhat more represented in this group than females. These two typologies are at the root of the very different patterns of MAFH consumed in rural and urban India shown in Fig. 9.

The heterogeneous nature of individuals’ and households’ FAFH experiences suggests that much of the consumption of FAFH is likely to be done outside of the purview of the key informant, underscoring the likelihood that it is under-reported. The relatively high average consumption levels and low intra-personal variability in school meals relative to purchased and other meals suggests the greater regularity of these particular types of MAFH.^8^ Measurement error in FAFH is most likely to be more problematic for the second typology of FAFH consumers; the 10% of Indians and the 40% of Indian households that either receive meals from “others” or purchase meals, especially in urban areas. These are the households that efforts to reduce measurement error in FAFH need to target.

With respect to the study’s first objective, we conclude that MAFH data can help to better understand FAFH and sources of measurement error. The discussion turns now to other sources of MAFH and FAFH data to address our second objective: the estimation of one component of measurement error in FAFH.

### 4.2 Household level-reported food (cooked meals) away from home in the last 30 days

We turn to the food consumption module household level reports based on the new food item categories. The discussion focuses on the three cooked meal-related questions. Table 2 presents the number of households reporting at least one of each of the three types of cooked meals, the total number and percentage share of each type and the conditional and unconditional averages.

**Table 2:** Number and composition of cooked meals reported in the food consumption module: reported apparent consumption of three specific types of cooked meal, India 2011/12. (Recall Period: 30 Days; 250,329,276 Households).

Type of Cooked Meal	Households Reporting >0		Total Cooked Meals		Average Quantities-Unconditional		Averages Quantities-Conditional	
	Number	Percent	Quantity	Percent	Mean	Median	Mean	Median
Purchased	2,74,83,449	11.0%	69,14,27,280	26%	2.76	0.00	25.16	12.00
Received free in workplace^a^	46,59,402	1.9%	14,23,92,656	5%	0.57	0.00	30.56	24.00
Received as assistance^a^	5,50,59,233	22.0%	1,86,36,24,555	69%	7.44	0.00	33.85	26.00

Total	8,18,82,018	32.7%	2,69,74,44,491	100%	10.78	0.00	32.94	25.00

Unconditional: Includes all households, those not reporting consuming any of the specific meal type.

Conditional: Includes only those households reporting consuming some of the specific meal type.

a Does not include cooked meals received from other households.

By far, the most important source of a cooked meal was from some form of assistance: it accounted for accounting for nearly 69% of all meals and 67% of households reporting having consumed one of the three types of cooked meals that were asked about. Purchased cooked meals comes in at less than half those levels and employer-provided free meals were relatively uncommon.

### 4.3 Juxtaposing the 2011/12 MAFH and FAFH estimates

A priori, one would expect that the Block 4 data would be more accurate due to its asking specifically about each individual household member’s behavior, rather than asking the respondent to recall all of the meals consumed away from home by all of the household members over the reference period. As noted earlier, answering household level questions is a more demanding cognitive task and is subject to greater memory and computational errors. Both approaches, however, rely on a single household respondent to provide information on all household members, which likely makes both approaches subject to measurement error since the respondent may simply not know about all of the meals all of the household members ate away from home. This is especially true when household members have stayed away from home for more than 24 h, which, as noted earlier, in India is highly correlated with the number of meals eaten away from home. In both approaches, the magnitude of the measurement error is likely to be highly correlated with household size owing to the increasing difficulty of tracking the behavior of larger numbers of household members. It is also likely to be correlated with other variables which are associated with increased consumption of food away from home, including more frequently staying away from home for 24 h or more, urban residence, source of meal, male, the age of the household members and other factors which are associated with increased consumption of food away from home, variables that were captured in the two distinct typologies of MAFH discussed earlier.

As noted in the methods section, the MFAH and FAFH measures have two identical meal source categories in common. Both include “free meals from employers (“free at the workplace”) and “paid” (“purchased meals”). As shown in Table 3, the two sets of estimates of employer and purchased meals are very similar both in terms of the number of meals and the percentage of households reporting having consumed one such meal.^9^ The FAFH estimates are slightly higher for both measures: 3% and 1%, respectively, in terms of the number of meals, and 0.1 and 0.5 percentage points, respectively, in terms of the percentage of households reporting having consumed one or more such meals.

**Table 3:** Juxtaposing numbers of meals away from home and food (cooked meals) away from home India 2011/12 (millions of meals in the last 30 days).

Measure	Employer	Purchased	School	Other Free Meals	Assistance	Meals Received from Other HHs	Total
*A. No. of Meals*							
MAFH	131	674	1792	1068			3664
FAFH	142	691			1864	845	3542
*B. FAFH as a % of MAFH*	109%	103%	95%				97%

*C.% of House-holds with* >*0*			35.7%				
MAFH	1.8%	10.5%	21.2%	17.1%			43.2%
FAFH	1.9%	11.0%			22.0%	13.7%	43.2%
					35.7%		

Comparing the two approaches’ other meal food sources is less straightforward. Table 4 presents a cross-tabulation of the number and percent of households with one or more MAFH and one or more FAFH. It shows that the results were the same for 92.2% of all households. The sum of the MAFH estimates of schools and other free meals totals 2923 million meals. This is 7.9% greater than the sum of FAFH’s sum of assistance and our Block 4-derived estimate of meals received from other households, which comes to 2709 million. Using either of the two approaches, the percent of households that consumed one or more meal other than an employer-provided or paid one are identical, 35.7%. The percent of households reporting consuming any MAFH or any FAFH are also identical: both are 43.2%. This suggests that the measures are remarkably close. Still, there is the possibility that these percentages reflect different combinations of households, and the question remains, how well do these two sets of estimates track one another?

**Table 4:** Concordance of household measures of FAFH and MAFH, India 2011/12.

A. Number and Percent of Households with One or More MAFH, One or More FAFH					
			*Total Meals Away from Home*		
			No	Yes	Total
*Total Food Away from Home*	No	Number of Households	14,18,16,340	1,92,69,404	16,10,85,744
		Percent of Households	56.7%	7.7%	64.3%
	Yes	Number of Households	3,37,465	8,89,06,068	8,92,43,533
		Percent of Households	0.1%	35.5%	35.7%
	Total	Number of Households	14,21,53,805	10,81,75,472	25,03,29,277
		Percent of Households	56.8%	43.2%	100.0%
Kappa Coefficient of Agreement: 0.837 (“Near perfect” agreement)					

B. Concordance of Total Quantity of MAFH and Total Quantity of FAFH

Pearson's r: 0.891 (Measure of precision)
C_b: 0.993 (Measure of accuracy)
Lin's concordance coefficient (rho_c): 0.886

We assessed the consistency of the pairs of estimates using concordance coefficients. This is a more rigorous analysis than simply looking at a Pearson correlation coefficient because concordance coefficients take into account not only the linear relationship between the two sets of estimates but also the magnitude of their differences; that is, it assesses both the bias and the precision of the pairs of estimates. We used Cohen’s Kappa index to assess the agreement between whether households were identified as having one or more meal away from home and one or more cooked meal (food) away from home. The estimated Kappa coefficient of agreement between the two measures is 0.837, indicating “near perfect” agreement (Tang et al., 2015).

We calculated Lin’s concordance correlation coefficient to assess the relationship between the pairs of estimates of the total quantity of MAFH and the total quantity of FAFH. The coefficient is the product of the Pearson’s r (a measure of precision) and the bias correction factor (a measure of accuracy), all three of which are shown in Table 4. The coefficient’s estimated value was high, 0.886.

We then calculated the same measures for the three disaggregated components of MAFH and FAFH to better understand the relationship between these two composite measures. The results are presented in Table 5. With a Kappa coefficient of 0.982, the Purchased-Paid variables track one another very closely in terms of identifying households with some consumption of paid meals. There is considerably greater variation in the paired quantities of these meals reported by households: Pearson’s r is estimated to be 0.633 and Lin’s concordance correlation coefficient is 0.576. Households’ reports of both consumption measures for Employer-Workplace track one another very closely, with Kappa and Lin coefficients of 0.961 and 0.978, respectively. The School & Others-Assistance measures have by far the greatest variability in households’ consistently reporting consuming one or more such meals, while the two quantity of meals measures of concordance are very similar to those of the Purchased-Paid variables’.

**Table 5:** Disaggregated concordance analysis of the component parts of MAFH and FAFH: percent of households consuming >0 and numbers of meals consumed.

MAFH Measure	FAFH Measure	Percent of Households Consuming >0: Kappa Coef.	Number of Meals Consumed	
			Pearson's r	Lin's Concordance Correlation Coef.
Purchased	Paid	0.982	0.633	0.576
Employer-Provided	Workplace-Provided	0.961	0.978	0.978
School & Others	Assistance	0.666	0.665	0.606

Others: other households, other government agencies and non-government organizations assistance: schools, other government agencies and non-government organizations.

We conclude that the measurement errors in both the percentage of households consuming at least one MAFH and one FAFH, and the quantities of MAFH and FAFH, as reflected in the differences in these estimates, are relatively small. In turn, we conclude that the disaggregation of the FAFH question and its closer correspondence with the MAFH questions enabled the more accurate responses reported in the household level questions. That does not mean to suggest, however (for reasons already noted), that measurement error in MAFH and FAFH in absolute terms are necessarily small (due, for example, to the limited awareness that a single household respondent may have of all household members’ eating behaviors when they are away from home, or the challenges a single respondent confronts in terms of memory lapse and arithmetic calculations).

### 4.4 Assessing the impact of the 2011/12 questionnaire changes: juxtaposing the comparative analyses of the 64th and 68th rounds

The discussion turns to an analysis of the variations in the pre- and post-questionnaire changes in FAFH and MAFH measures in an attempt to better understand the impact of the modifications in the questionnaire. In light of the many potential sources of measurement error (Biemer et al., 2004, Bradburn et al., 2004, Carroll et al., 2015) this should not be regarded as a definitive analysis of the existence of measurement error or of its causes. What is presented here is best regarded as a plausibility analysis of the impact of the modifications in the questionnaire that quantifies the general order of magnitude of the differences in the 2007/08, Round 64, FAFH and MAFH estimates and those in the 2011/12, Round 68 (Habicht et al., 1999). Recall, whereas in both rounds the MAFH-related questions were identical, Round 64’s estimate of FAFH was based on just two food item entries “cooked meals,” that were unpacked in Round 68 into five sub-categories. We focus on a subset of three of the Round 68 entries, those directly comparable to the MAFH categories.^10^

**Fig. 10:**
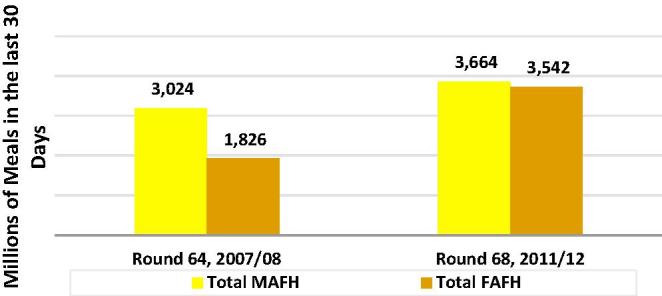
Comparing the two rounds' estimates: individual-level MAFH vs household-level FAFH.

As is evident in Fig. 10, there have been huge changes over the two Rounds’ in the estimates of MAFH and FAFH. The considerable disparity in the two pairs of estimates in Round 64, were largely eliminated in Round 68. Total FAFH estimates went from the equivalent of just 60.4% of total MAFH estimates, to 96.7%; virtually eliminating this source of FAFH measurement error.

We again conducted concordance tests of each household’s pairs of estimates and found the Kappa coefficient of each household’s consuming at least one MAFH and its consuming at least one FAFH to be substantially lower (0.6111, P < 0.056) than the post questionnaire changes in Round 68. The Lin concordance correlation coefficient of the consistency of the reported quantities of MAFH and FAFH was also much lower (0.622, Pearson’s r was 0.627, and the bias-correction factor was 0.993). The results underscore our conclusion that the modifications in the questionnaire introduced in 2011/12 have significantly reduced measurement error in FAFH in India.

**Fig. 11:**
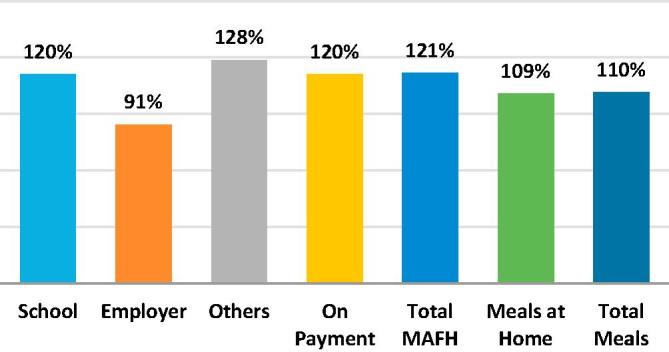
Changes in the types of meals consumed in the last 30 days, India 2007/08–2011/12 Round 68 as a percent of Round 64.

**Fig. 12:**
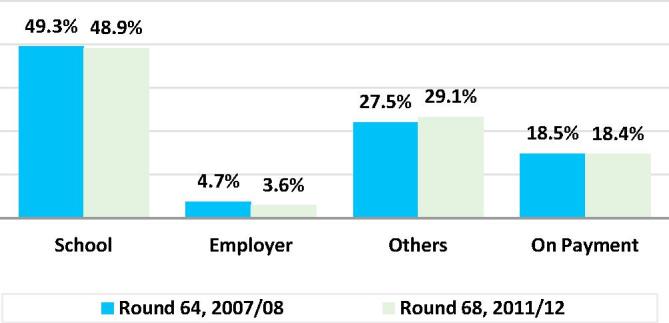
Changes in the composition of meals away from home (last 30 days), India 2007/08–2011/12.

The meals data also allows us to see changes in the number and composition of meals over time, thereby providing some insight into how much larger FAFH-related measurement error might have been had there been no changes in the questionnaire, while also allowing us to better understand the recent dynamics of FAFH. As may be seen in Fig. 11, with the exception of Employer meals, all of the Round 68 estimates of different types of meals reported in the individual level questions were greater than their Round 64 levels. Fig. 12 shows how the meal types rates of change varied over the two rounds. The shares of School and On Payment remained about constant, both falling by less than 1%, while those of Others grew by 6% and that of Employer fell by 24%. It may be inferred that without the changes introduced in the 2011/12 questionnaire, measurement error would have been significantly higher in 2011/12 relative to 2007/08.

## 5 Discussion

The Indian’s introduction of measures tying FAFH at the household level to MAFH at the individual level has lessons for other countries. The more unique and critical element of the approach is the set of seven, comprehensive, mutually exclusive, meal pattern-related questions that together constitute a conceptual framework that helps survey interviewees and interviewers, alike, to better ensure the consistency and reliability of responses to FAFH questions. It requires respondents to think through and report each individual household member’s usual pattern of meal consumption during the reference period, thereby providing a benchmark to better ensure the consistency and reliability of responses to FAFH questions, as well as well as a tool for shaping modifications or additions to the food item list.

The HCES of most countries do not currently have a similar set of individual household member level questions about their meal pattern that can be linked directly to FAFH items during the same reference period. Most countries have, instead, only a handful of questions about food items that ask about FAFH at the household level. As a result, the cognitive task required of the single household respondent is far more complex, and, no doubt, at the root of much of the measurement error associated with FAFH in most HCES. The respondents are asked to recall (although many probably use instead, rate-based estimation methods) the number of meals each individual member of their household had for each of the two or three meals they ate each day of the reference period for each day of the reference period. That is a challenging task that involves potentially many different calculations for each individual that must then be summed over all members of the household. Moreover, it is not done just once, but once for each of the food list items involving FAFH.

In contrast, the MAFH-linked approach facilitates the cognitive process of developing these estimates by providing a tool with which to break down the recalling (estimating) process into several steps, making it more manageable, and a less memory- and arithmetically-demanding activity, and is likely to yield results that are considerably more accurate.

This study demonstrates that combining information of individual-specific meal data that can be linked with the HCES food consumption module’s food list items can capture a substantially larger quantity of FAFH, which we infer constitutes a significant reduction in the measurement error in FAFH. We believe that this source of measurement error—which has grown over time with FAFH—has been one important contributing factor in the apparent falling caloric intake of Indians over time. By reducing the magnitude of this under-reporting of consumption, the Round 68 questionnaire revisions have reduced the impact of one factor that has contributed to the apparent paradox. While this has reduced the magnitude of the apparent paradox at the heart of the great calorie debate, this study makes no quantitative claims about how much these changes may have reduced the size of the under-reporting of caloric intakes. A crude, first approximation might be gained by assuming it to be equal to the difference in the discrepancies between MAFH and FAFH in Rounds 68 ad 64, and computing that as a proportion of all FAFH. That comes to about 2% of all meals; not an enormous part of the puzzle but a portion of it.

The analysis of MAFH has generated a number of insights: (1) MAFH consumption patterns are highly segmented—persons who had at least one MAFH generally consumed one and only one type of MAFH; (2) individual and household MAFH consumption patterns were quite different, reflecting high within household variability; (3) there is lower intra-person variability in school meal consumers compared to purchased and “other meals” consumers; and (4) MAFH consumers in India are largely captured by two typologies; one consisting of rural children 5–15 consuming school meals, the second comprised of urbanites 16 years of age and older who consume predominantly purchased and other meals, with somewhat more other meals.

These findings suggest that current methods used to estimate the importance of FAFH—including the use of a single nationwide average cost per nutrient (e.g., Tandon and Landes, 2011), or a single nationwide coefficient of variation (Sibrián et al., 2015) to develop estimates of the nutrient availability or apparent nutrient consumption—are too simplistic; too aggregative, do not take advantage of all available information and are likely to be an important factor contributing to the sensitivity of food security measures to alternative estimation methods. These approaches need to be improved upon: more of the available information about intra-household variations in meal and food consumption patterns needs to be used in analyzing food availability and insecurity.

While the Round 68 questionnaire revisions do not provide definitive solutions to the challenge of estimating the caloric (and other nutrient) content of meals, snacks and other processed foods for which only values—and not quantities—of foods are collected, by providing more detailed information about meals eaten, they provide opportunities for improving these measures. For example, by combining the individual-based meals data from the MAFH module with the household-based FAFH data we are able to better measure which members of the household are eating school-provided cooked meals, and combining this information with the national standards of the Mid-Day-Meal Scheme’s minimum dietary requirements (Table 6), and assuming those standards are met^11^, we can do a better job of estimating the nutrient contribution of school meals (49% of all MAFH) consumed by households and individual household members.

**Table 6:** Minimum mid-day-meal program caloric and nutrient content of the meal served per child.

Item	Primary School	Upper Primary School
1. Calorie	450	700
2. Protein	12	20
3. Rice/wheat	100 Grams	150 Grams
4. Dal	20 Grams	30 Grams
5. Vegetables	50 Grams	75 Grams
6. Oil & fat	5 Grams	7.5 Grams

It is worth noting that although the discussion here has focused only on meals, another, related improvement in the Round 68 questionnaire was the introduction of cooked snacks (food item 283). Earlier rounds contained no food item for cooked snacks, although they may have been partially captured in “other processed food” (food item 308 in Round 64) and perhaps elsewhere.^12^

The NSS interviewer field guidelines have long attempted to distinguish between meals and snacks, and yet the questionnaires prior to Round 68 have not included any explicitly identified item to capture snacks. With the introduction of the three types of cooked meals and a cooked snack variable we expected to find the snack variable to relatively quantitatively insignificant. We were surprised to find that it accounted for 25.1% of the total value of the sum of the three cooked meals and cooked snack values. We hypothesize that the introduction of “cooked snacks” may have been another source of reduced measurement error—in particular, the under-reporting of consumption—compared with earlier rounds.^13^

The findings of this study also suggest that many of the unknowns about FAFH and likely sources of measurement error are likely to be attributable to the persons who eat purchased meals and other meals in urban areas. This is where additional efforts to better understand FAFH and reduce measurement error needs to focus. Specifically in the case of India, consideration should be given to prioritizing this typology of consumers with 24HR and other surveys to better understand FAFH and to further reduce FAFH-related measurement error.

In a recent study analyzing Bangladesh data, D’Souza and Tandon (2014) found that 36% of individuals in food insecure households were food secure, and that 32% of individuals in food secure households were food insecure. To be sure, this is only one survey and only one country, but the finding is consistent with the evidence found here as well. Clearly, to better measure and better understand food security we need to know what is happening within households. In most countries, that will require unpacking some of the household level data collection, which—given the multi-purpose nature of HCES and the large number of already-existing HCES stakeholders, will likely be something that is likely to require some time to do and which may only be possible to implement slowly, incrementally and selectively.

HCES analysts, and more generally, the international development and food security communities, need to take greater advantage of the natural laboratory provided by the heterogeneous body of existing HCES surveys to harvest more lessons—like these meal pattern insights from India—to better understand alternative questionnaire design options and the tradeoffs they involve. This should be part of a global process that works to share information and harmonize the design of HCES around approaches that will help to reduce measurement error in FAFH and other indicators. Clearly one size does not fit all. Moreover, the requirements to generating better survey data that are proposed here—viz., the addition of new questions and modifications in select existing questions—does not come without its own costs and tradeoffs. They entail disruptions in the time series of HCES variables which concomitantly puts an end to, or at minimum, compromises the ability to conduct inter-temporal analyses of the variables that are modified. Still, without change, the many HCES that currently do not do an adequate job collecting data on FAFH risk losing their relevance, and with it a potentially important source of evidence for better understanding and providing input into food and nutrition policymaking and programming. Consideration should be given to devising a strategy for making suggestions not only of adding additional questions, but for cutting or simplifying questions as well. Some initial possibilities might be identified by reviewing the frequency and value of each of the items on the food list, or, if there are currently questions about meal partakers, consideration should be given to dropping them as they will be rendered largely superfluous by the comprehensive, 7-question, individual-level meals section.

## 6 Summary and conclusion

FAFH is important and it is becoming more important. The Indian experience suggests that combining the introduction of specific, common FAFH items in an HCES food list and collecting individual household member level data on broad categories of meals by type and source, can together significantly improve more accurately capturing FAFH. Collecting data on meals can help to reduce measurement error by helping to ensure more comprehensive and complete reporting of FAFH, and greater consistency in how food and meals are consumed away from home. While 90% of HCES now include some FAFH items in their food lists, few collect data on meals in a way—like the Indians—that attempt to establish recent eating patterns such that it can provide a frame of reference to improve the relevance and reliability of HCES in measuring food consumption. The addition of the 7 meal-related questions the Indians have been asking for 50 years should be considered by more countries. This is a starting point for strengthening many HCES. Important, unaddressed, questions still remain: Can the general Indian food list item categories be improved upon? Can they be made more telling in terms of nutrient content? The work of re-purposing HCES to provide more and better evidence for designing food and nutrition policymaking is just beginning.

## Appendix A

### A.1 Lin's concordance correlation coefficient

To create an index of concordance scaled to between −1 and 1, use:

$$\begin{array}{c} \text{CCC}=1-\frac{\text{E}\left[{\left(\text{Y}-\text{X}\right)}^{2}\right]}{\text{E}\left[{\left(\text{Y}-\text{X}\right)}^{2}|\rho =0\right]}\\ \;\;=1-\frac{{\left(\mu_{\text{y}}-\mu_{\text{x}}\right)}^{2}+\sigma_{\text{y}}^{2}+\sigma_{\text{x}}^{2}-2\rho \sigma_{\text{y}}\sigma_{\text{x}}}{{\left(\mu_{\text{y}}-\mu_{\text{x}}\right)}^{2}+\sigma_{\text{y}}^{2}+\sigma_{\text{x}}^{2}}\\ \;\;=\frac{2\rho \sigma_{\text{y}}\sigma_{\text{x}}}{{\left(\mu_{\text{y}}-\mu_{\text{x}}\right)}^{2}+\sigma_{\text{y}}^{2}+\sigma_{\text{x}}^{2}}\\ \;\;=\rho \left\{\frac{2}{\frac{{\left(\mu_{\text{y}}-\mu_{\text{x}}\right)}^{2}}{\sigma_{\text{y}}\sigma_{\text{x}}}+\frac{\sigma_{\text{y}}}{\sigma_{\text{x}}}+\frac{\sigma_{\text{x}}}{\sigma_{\text{y}}}}\right\}\\ \;\;=\rho \left\{\frac{2}{\nu^{2}+\omega +1/\omega }\right\}\\ \;\;=\rho X_{a} \end{array}$$

where

$$X_{a}=\frac{2}{\nu^{2}+\omega +1/\omega }$$

$$\omega =\frac{\sigma_{\text{y}}}{\sigma_{\text{w}}}$$

$$\nu =\frac{\left|\mu_{\text{y}}-\mu_{\text{x}}\right|}{\sqrt{\left(\sigma \sigma_{{\text{x}}^{\prime \prime }}^{\prime \prime }\right)}}$$

The quantity ω is the scale shift and the quantity ν is the location shift relative to scale.

The correlation ρ is a measure of precision and the quantity X_a_ is a measure of accuracy.

Cohen's Kappa is the normalized chance-adjusted agreement between two series of binary values:

$$\kappa =\frac{\rho_{\text{o}}+\rho_{\text{e}}}{1-\rho_{\text{e}}}=1-\frac{1-\rho_{\text{o}}}{1-\rho_{\text{e}}}$$

where *p_o_* is the relative observed agreement between the series, and *p_e_* is the hypothetical probability of chance agreement, using the observed data to calculate the probabilities of each series randomly identifying each category.

If the series are in complete agreement then κ = 1.

If there is no agreement among the series other than what would be expected by chance (as given by pe), κ ≤ 0.

Sources: (Cohen, 1968, Lin, 1989, Tang et al., 2015).

## Footnotes

3 Measurement error occurs for a variety of reasons, some of which are attributable to the interviewee, others to the interviewer and still others to the questionnaire or data collection method (Biemer et al., 2004, Bradburn et al., 2004, Carroll et al., 2015).

4 This is not to suggest that the data collected on MAFH have never been analyzed before. They have been analyzed in several papers (Deaton and Subramanian, 1996, Tandon and Landes, 2011).

5 The NSSO Instruction to Field Staff guidelines notes: “A meal will be considered to be taken at home if the meal is prepared at home irrespective of the place where it is consumed” (NSSO, 2012: Chapter Three, paragraph 3.4.12). It also notes that if “school/*balwadi*’s etc.” (column 10 of the schedule) are “received free it will be recorded in column (10) Meals received at subsidized rate will be recorded in column (13)” (NSSO, 2014: Chapter Three, paragraph 3.4.14). (This suggests that the common, ambiguous appellation “food away from home” would be more accurately labeled as “food that is purchased and consumed away from home”.)

6 This is designed to avoid double counting meals–i.e., counting meals from both the perspective of the household providing the food and from the perspective of the household receiving the food.

7 The measure does not include cooked snacks or other processed (but not cooked) foods or snacks consumed away from home—both of which are items in the revised food list.

8 While the relative standard error (RSE) of all of the FAFH and MAFH measures are low and well within the conventional range of acceptability (<25–30%), the RSE of the unconditional mean of “other meals” and “purchased meals” are 173% and 194%, respectively, that of “school meals,” and the RSE of their conditional means are 261% and 267%, respectively, that of “school meals.”

9 For employer meals, 99.86% of the two measures’ pairs of estimates of households having one or more meal-food employer were identical, either yes or no. For paid meals, 99.65% of the pairs were identical.

10 It should be noted that Round 64 was a “thin” round and as such, was not specifically designed as a (total) expenditure survey. With the exception of the changes introduced in the food list’s FAFH items, however, it included the identical food list as Round 68, and thus for our purposes the survey instruments are comparable. Moreover, the two rounds used the same sampling frame, and while the sample size of Round 64 is only about 35% of that of Round 68, its sample size is nevertheless sufficiently large (50,927 households) to provide adequately precise estimates for this comparative analysis.

11 This approach is the equivalent of what is referred to as the “intention-to-treat” approach in program evaluation literature.

12 Snacks are included in other items as well—especially in the category of packaged processed foods (food list items 290–296), which includes sweets, cake, pastry, biscuits, chocolates, chips, papad, bhujia, namkeen, chanachur.

13 By providing specific examples of the types of snacks the new question is intended to capture (papad, bhujia, namkeen, chanachur), it provides an opportunity for quantifying the significance of these snacks beyond their value; for example by developing an average caloric content per unit of the identified items.
